# Long-term efficacy assessment of current treatment options for epistaxis in HHT

**DOI:** 10.1007/s00405-021-06701-z

**Published:** 2021-03-04

**Authors:** Cilgia Dür, L. Anschuetz, S. Negoias, O. C. Bulut, A. Angelillo-Scherrer, M. Caversaccio

**Affiliations:** 1 Department of Otorhinolaryngology, Head and Neck Surgery, Inselspital, University of Bern, Bern, Switzerland; 2 Department of ENT, Head and Neck Surgery, University of Basel, Basel, Switzerland; 3 Department of ENT, University Heidelberg, 69120 Heidelberg, Germany; 4 Department of Hematology, Inselspital, University of Bern, Bern, Switzerland

**Keywords:** Rhinology, Epistaxis, Hereditary hemorrhagic telangiectasia, Rendu-osler, Laser photocoagulation, Bevacizumab

## Abstract

**Purpose:**

Hereditary hemorrhagic telangiectasia (HHT) is a vascular disorder that presents with recurrent, intractable epistaxis. The aim of this study was to retrospectively analyze the efficacy of various treatment options for epistaxis in patients with HHT, over a period of 18 years, and to correlate these findings with available evidence in the literature.

**Methods:**

Records of patients with HHT, treated for epistaxis between 2000 and 2018 were analyzed. Treatment procedures carried out and their efficacy were extracted and analyzed.

**Results:**

Forty-three records were evaluated. All patients were given nasal humidifying ointments, 93% required acute treatment with bipolar electrocautery, and 60% underwent atraumatic nasal packing. Recurrent cases were treated medically with tranexamic acid (26%), oestrogen (19%), and bevacizumab (2%). Laser photocoagulation was done in selected cases (40%) and if unsuccessful, septal dermoplasty was performed (2.3%). Endovascular embolization was reserved for life-threatening emergencies (7%).

**Conclusion:**

Epistaxis in HHT is not curable, but can be managed by employing a comprehensive stepwise approach. An algorithm for effective and comprehensive management has been presented.

## Introduction

Hereditary haemorrhagic telangiectasia (HHT), also known as Osler–Weber–Rendu disease, is a rare autosomal dominant disorder causing angiodysplasia. It occurs in one of every 5000 persons, and is believed to be underdiagnosed [1]. In HHT, capillaries of the skin, mucous membranes, and specific organs such as lungs and liver are absent, and there is direct communication between the dilated arteries and veins. This causes extreme fragility of the vessels, which can rupture at even the slightest trauma. This is clinically visible as mucosal or cutaneous telangiectasias and arteriovenous malformations.

Epistaxis is the most common clinical manifestation of HHT. At least one episode occurs by 12 years of age in most patients, often before the disease has been diagnosed. It occurs in almost all patients by 40 years of age. More than 50% of patients suffer from recurrent epistaxis before the age of 20, which becomes more severe in later decades. By the age of 40, epistaxis frequently occurs in almost all patients with HHT [1]. The frequency and severity of epistaxis often leads to iron deficiency anaemia, requiring multiple blood transfusions. This has a negative impact on the quality of life [2].

Several treatment options have been described in the literature [3–9]. Medical modalities of treatment range from simple humidification to use of complex drugs like thalidomide [3, 4] and bevacizumab [5, 6]. Surgical options have, included septal dermoplasty [7], endovascular embolization [8], and even complete nasal closure [9]. None of the treatment methods have proved to be complete solutions. Resistance has been noted to several medical treatments, and surgical treatment does not necessarily improve quality of life. Therefore, the ideal treatment option has yet to be identified.

With this background, we aim to add the existing body of literature on the management of epistaxis in HHT by retrospectively analysing different treatments carried out for epistaxis in HHT patients. We attempt to correlate these findings with literature evidence to identify the best protocol to be followed in such patients.

## Methods

This study was approved by the institutional and regional review board (Inselspital, University Hospital Bern, Bern, Switzerland, KEK-No. 201-00743). Owing to the retrospective nature of the study, formal written informed consent was not required.

The data of all patients treated in the ENT department of the University Hospital in Bern between 2000 and 2018 were retrospectively analysed. Records of patients who appeared to have HHT were included for analysis. Patients were considered to have HHT if they had satisfied at least 3 of 4 of the Curaçao criteria [10] (Table 1).

**Table 1:** Curaçao’s criteria for HHT diagnosis

Features of HHT
Spontaneous recurrent epistaxis
Multiple characteristic telangiectasias
Visceral AV malformations
First degree family member who has HHT

Source: Shovelin et al. [10]

0–1 features—unlikely; 2 features—suspected; 3–4 features—definite diagnosis

The information extracted from the records, included clinical notes from consultations, operations, and radiographic examinations. Epidemiological data and the presence of anaemia and need for transfusion, septal perforations, screening for arteriovenous malformations, and genetic testing were noted. The various medical and surgical treatment modalities employed were recorded for each patient as well as the time to bleeding following each treatment modality. The extent of follow-up after treatment was checked.

## Results

A total of 51 records of patients suffering from HHT were obtained. Complete records were available for 43 patients, and these were analysed. Of these 43 patients, 22 were male (51%) and 21 female (49%). The average age at diagnosis was 45 years. Among these patients, 28 (65%) suffered from iron deficiency anaemia due to chronic bleeding. Owing to the anaemic status, 17 patients (40%) required blood transfusions at least once.

### Acute episodes

All 43 patients had visited the emergency department at least once due to severe epistaxis. Acute management of epistaxis was performed in 40 patients (93%) using bipolar electrical coagulation. Twenty-six patients (60%) underwent nasal packing using lubricated and ensheathed packs. Episodes leading to hospitalization were present in 24 patients (56%). Three patients (6.9%) underwent embolization, and one of them (2.3%) received septal dermoplasty.

### Long-term management

For prevention of bleeding in the long term (chronic management), conservative medical management was tried first. If that was not successful in terms of reducing the frequency and severity of bleeding events, then surgical modalities were employed.

### Conservative medical management

All patients were advised to use hydrating therapy to keep the nasal mucosa humidified. This included saline or lanolin and dexpanthenol ointments. Eight patients (19%) received oestrogen-containing ointment. One patient (2%) was treated with a locally administrated bevacizumab. Eleven patients (26%), who had developed anaemia due to chronic bleeding, were prescribed oral doses of tranexamic acid ranging from 500 to 1000 mg, one to three times a day.

### Surgical therapy

In patients who did not respond to medical management, surgical treatment was carried out. Laser photocoagulation was performed in 17 cases (40%). Of these, 13 patients (30%) underwent a Nd:YAG laser treatment and four patients (9%) underwent Argon or CO2 laser treatment. Seven of the laser-treated patients (16%), who required two or greater than two sessions, were considered as repeat patients. Of these, six patients required repeat Nd:YAG laser, and one required repeat Argon laser treatment. Four patients (10%) developed septal perforation.

In the three patients who underwent embolization, the facial, maxillary, or sphenopalatine artery was addressed. Two patients required this procedure more than once. One of these patients, who underwent embolization three times, had been receiving bevacizumab therapy previously. The other patient had previously received cautery and three rounds of laser photocoagulation, which was unsuccessful, and subsequently underwent transarterial embolization of facial, maxillary, and sphenopalatine arteries. He later also received septal dermoplasty and local radiotherapy.

None of the above therapeutic interventions were able to completely eliminate bleeding episodes. The duration of reduction of the need for hospital visits (both emergency and planned visits), due to a severe bleeding episode ranged from none to up to 4 years. The time to hospital visits due to bleeding episodes was compared for different modalities of treatment using the Kruskal–Wallis test. The values were found to be significant (H statistic 12.889, *p* = 0.004).

**Fig. 1:**
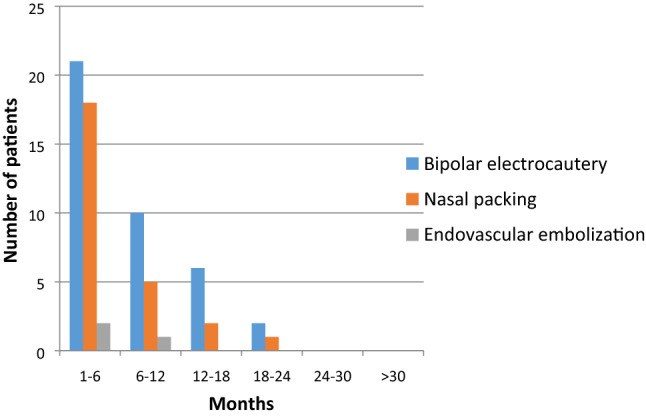
Time to re-bleeding following the treatments for acute bleeding

**Fig. 2:**
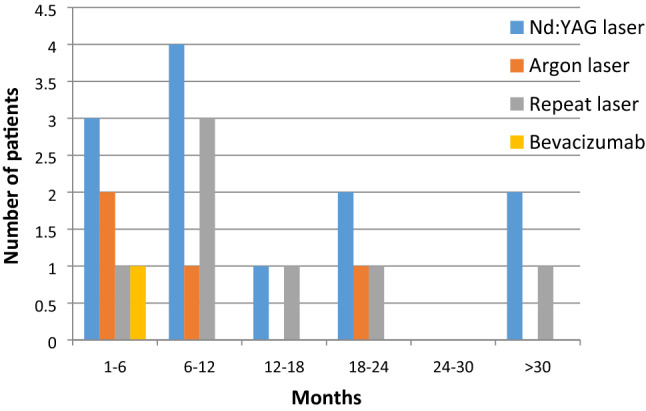
Time to re-bleeding following the treatments for chronic recurrent bleeding

Figures 1, 2 illustrates the time to bleeding episodes after acute and chronic treatment options were employed. Most effective results were obtained with Nd:YAG laser, either with single or repeat procedures (Table 2).

**Table 2:** Average time to hospital visit due to bleeding with various forms of treatment

Treatment modality	Number of interventions	Mean time till bleeding (months)	Standard deviation
Treatments for acute bleeding			
Bipolar electrocautery	39	7.4615	5.8076
Ensheathed packs	26	6.0385	5.1574
Embolization	3	6.6667	5.0332
Treatments for chronic recurrent bleeding			
Nd:YAG laser	13	16.6154	12.7249
Repeat Nd:YAG	6	14.667	13.35
Argon laser	4	10.25	7.2284
Repeat Argon	1	24	–
Bevacizumab	1	1	–

### Adjunctive treatment

Twelve patients (28%) underwent screening in our hospital for arteriovenous malformations (AVMs). Seven patients (16%) were found to have pulmonary AVMs and one patient (2%) had a cerebral AVM. One patient, who had juvenile polyposis and HHT (2%), was genetically tested and found to have SMAD4 gene mutation.

## Discussion

This retrospective study illustrates a variety of options for treating epistaxis in HHT patients, representing, to our knowledge, the biggest HHT patient cohort in Switzerland so far. The present study shows that despite numerous options for treatment, epistaxis continues to be a recurrent problem in HHT patients. The medical literature provides support for the treatment modalities employed as well as for other forms of treatment. We have reviewed all the methods used to manage epistaxis in HHT patients described in the literature, and compared this to the protocols used in our cohort.

### Conservative strategies for management of epistaxis

For acute management of bleeding, the only conservative strategy that can be employed is nasal packing. Nasal packing is a safe option when bilateral bleeds are present; unlike coagulation methods, there is no risk of septal perforation. In the current cohort, this was the preferred method for acute management of bilateral bleeds. The main disadvantage of nasal packing is that their removal at a later date can cause re-bleeding. This may be avoided by using pneumatic packs or sheathed standard packs. Pneumatic packs allow deflation prior to removal, and sheathed packs have a lubricated outer covering, which can minimize friction and trauma [11].

For reducing the frequency and duration of episodes, humidifying agents remain the first line of treatment for epistaxis in HHT patients. These agents can maintain integrity of the nasal mucosa. They prevent nasal crusting and reduce damage to the endonasal mucosa, leading to a reduction of epistaxis [11]. One observational study found that using sesame/rose geranium oil showed good efficacy in reducing the Epistaxis severity score. This was attributed both to nasal hydration, and formation of a protective layer [12]. Nasal ointments usually contain lanolin, dexpanthenol, or saline. However, there is a lack of sufficient data to recommend one topical therapy over another. In the current cohort, humidification was prescribed as the first line treatment for all patients.

### Medical therapy for management of epistaxis

Hormonal therapy, including oestrogen and anti-oestrogen therapy with tamoxifen, has been used in the past for management of epistaxis in HHT. Early anecdotal reports suggested that high-dose oestrogen could decrease epistaxis frequency [13]. However, a randomized controlled trial by Vase et al. indicated that there was no significant difference in bleeding between the oestrogen and control group [14]. On the other hand, tamoxifen has proven to be successful in decreasing the frequency of epistaxis, in case reports, as well as in a randomized controlled trial [15, 16]. In our cohort, eight (19%) patients received oestrogen-containing ointments that were applied topically.

The use of haemostatic agents has also been used to stop bleeding episodes. Tranexamic acid, an anti-fibrinolytic agent, has been used to treat HHT patients. Geisthoff et al. conducted a double-blinded trial evaluating the effect of tranexamic acid against placebo [17]. They found that while this drug reduced the frequency of epistaxis, the haemoglobin levels did not change significantly. In our cohort, 11 patients (26%) received oral treatment with tranexamic acid. In patients who are at high risk of developing thromboembolic events, local administration of tranexamic acid may be considered. In this cohort, two patients (9.5%) received topical application of 5 ml of tranexamic acid nose cream, in a concentration of 5 mg/ml.

Propranolol, a beta-blocker, is believed to have an anti-angiogenic effect. It reduces VEGF expression, and induces apoptosis of endothelial cells. Mei-Zahav et al. retrospectively analysed six patients who underwent treatment with topical propranolol gel (1.5%), twice daily for 12 weeks [18]. They found a significant improvement in epistaxis severity score and haemoglobin levels. The number of blood transfusions decreased, but not significantly. Contis et al. also analysed the effect of propranolol, taken orally (80–160 mg daily), in 21 HHT patients. They found that it decreased the intensity and frequency of episodes [19]. Timolol is another beta-blocker with similar actions. Ichimura et al. prospectively analysed the effect of topical timolol in 11 patients who had already undergone nasal dermoplasty. They found marked reduction in the intensity and frequency of bleeding [20]. In the current cohort, we did not use beta blockers, owing to the paucity of evidence on the subject at the time of treatment.

The advent of drugs that inhibit vascular endothelial growth factor marked an important step in the management of epistaxis in HHT patients. Bevacizumab, a recombinant form of monoclonal antibody, binds and inhibits VEGF, and thus prevents endothelial cell proliferation and angiogenesis. However, in a randomized trial, Whitehead et al. found that topical bevacizumab was no different than placebo in decreasing frequency of epistaxis [21]. Steineger et al. analysed intranasal submucosal injections of bevacizumab in 33 patients [6]. They found that although this treatment was effective, it was common to develop resistance to this drug at a later stage. Halderman et al. conducted an evidence-based review of eleven articles that analysed the efficacy of this drug in HHT patients [5]. They found that while topical administration was ineffective, submucosal and intravenous forms appeared to have some effect in controlling epistaxis. On the other hand, Stokes et al. systematically reviewed thirteen studies that reported either topical or submucosal bevacizumab therapy. They stated that both submucosal and topical bevacizumab therapy did not have any effect on frequency and duration of epistaxis [22]. In the current cohort, one patient who had recurrent epistaxis was placed on submucosal injections of bevacizumab. In accordance with published studies, it was not effective and re-bleeding occurred within a month. However, a recent literature review has supported the use of intravenous bevacizumab for patients with severe epistaxis. This was not attempted in our case series; but, based on evidence from the literature, it may be considered as a treatment option [23].

Pazopanib is another VEGF inhibitor that targets the enzyme tyrosine kinase. Not much has been reported regarding the use of this drug in HHT. Parambil et al. have reported the use of this drug in a 61-year-old male with HHT refractory to bevacizumab. The patient showed improvement in the severity of epistaxis with pazopanib, taken at 50 mg/day [24]. The dose was increased and maintained at 100 mg/day. This is a new drug and can be tried in the future for HHT patients who become resistant to bevacizumab.

Thalidomide is also known to inhibit angiogenesis. Fang et al. evaluated its use in seven HHT patients who had refractory recurrent epistaxis. Patients were evaluated using the epistaxis severity score (ESS), and they showed improvement in symptoms one to three weeks after the treatment had started. However, there were several side effects, including dizziness, drowsiness, nausea, constipation, and peripheral neuropathy [3]. Baysal et al. also evaluated its use in six patients [4]. They found improvement in both ESS and SF-36 scores, the latter evaluates quality of life. They did not observe notable side effects. Thalidomide was not used in the current cohort.

### Surgical and interventional therapy for management of epistaxis

When conservative approaches and medical therapy fail, several minimally invasive as well as open surgical procedures are available to reduce the frequency and severity of epistaxis in HHT patients. In the current study, this was required in 40% of patients when medical management failed.

For acute management of epistaxis, coagulation of blood vessels is the preferred method. This may be achieved either by bipolar cautery, or laser photocoagulation. Endonasal coagulation with bipolar cautery is immediately effective and seals off the ruptured vessel immediately. Ghaheri et al. reported its use in 47 patients, and they stated that it was effective in controlling epistaxis, either as a standalone method, or as an adjunct to laser coagulation [25]. In the present cohort, bipolar cautery was used for management of acute unilateral bleeds only, to minimize the risk of septal perforation. However, we found that the use of bipolar cautery was associated with faster return to the hospital with nasal bleeds.

Laser photocoagulation can destroy defective blood vessels with minimal injury to the surrounding normal mucosa [26]. It causes scarring and fibrosis, which lead to a decrease in telangiectasias. The effect is often time-limited and the procedure has to be repeated. Several laser devices with different wavelengths (ND:YAG, KTP, Argon, CO2) can potentially be employed. Abiri et al. reviewed fifteen studies that had employed different kinds of lasers for controlling epistaxis in HHT patients. They concluded that Nd:YAG and argon lasers showed better results as compared to argon lasers. For severe epistaxis, Nd:YAG lasers were superior to argon lasers [27]. In the present cohort, more favourable long-term results were achieved with the use of ND:YAG laser. Although this was preferred used in patients because of its greater depth of penetration, ND:YAG laser does have its drawbacks. It cannot be used to control acute bleeding, and bilateral coagulation carries the risk of septal perforation. Septal perforation was seen in four patients in this study, but in these cases, the risk could have been potentiated by previous remedies such as bipolar cautery.

Radiofrequency coblation is an acceptable alternative to laser coagulation. This breaks down molecules into inert gases at comparatively lower temperatures, so the risk of thermal injury to tissues is less. Joshi et al. used coblation in five patients and found that it was safe, effective and well-tolerated [28]. Luk et al. also found that coblation was an effective alternative to KTP lasers, and subjects who had coblation experienced less nasal obstruction following the procedure [29].

A few studies have evaluated the effect of endovascular embolization. One study by Trojanowski et al. showed a success rate of 85%, but patients also had a high recurrence rate of 43% [8]. This therapy is mostly indicated in acute conditions. In the current study, we reserved this procedure for acute, life-threatening cases not responding to nasal packing. Our patients underwent endovascular embolization of the sphenopalatine and/or facial and/or maxillary arteries on one or both sides. Most authors do not recommend embolization, as it carries a high risk of stroke and blindness, particularly if the anterior and posterior ethmoidal arteries are treated at the same time.

External radiotherapy was tried in one patient in the present cohort, who was found to be refractory to most forms of treatment. Both external beam radiotherapy and brachytherapy can irreversibly destroy fibrodysplastic microvessels, thereby reducing epistaxis. Patients who underwent brachytherapy had favourable results with some patients experiencing complete remission [30]. External radiotherapy also seems to be effective and has been used in palliative settings after other treatment options have failed [31]. Both methods are associated with a risk of septal perforation. There is also a concern that radiotherapy might increase the risk of neoplastic transformation of cells.

Sclerotherapy has been used in two studies in the literature. Injection of sodium tetradecyl sulphate into defective vessels can cause scarring and fibrosis and reduce telangiectasias, and therefore, epistaxis. One trial, by Boyer et al. showed that sclerotherapy was more effective than several other standard forms of therapy [32]. Another observational, cross sectional study was carried out by Esteban-Casado et al. [33]. Thirty-eight patients underwent sclerotherapy with polydocanol, and received long-term topical nasal propranolol thereafter. Significant improvements were found in both the mean epistaxis severity score and quality of life. Further comparative studies are needed to validate this treatment option.

Septal dermoplasty is a procedure which is usually used only as a last line of defence. In septal dermoplasty, the nasal mucosa is replaced by skin or buccal mucosal graft tissue, while preserving the underlying perichondrium. Harvey et al. showed that septodermopasty can reduce the need for multiple laser procedures by as much as 57% [7]. However, telangiectasias have been found to grow through the skin graft, leading to recurrence of epistaxis. This procedure can also cause long-term nasal crusting, compounding the problem. Therefore, this procedure is more useful in severe cases. Nevertheless, the indications have not been clearly established in the literature. In the current study, we used this as a last resort, only when laser treatment had failed at least three times. In a previous study by Lesnik et al., the procedure was used in patients who had developed septal perforation following other treatments. They noted that the procedure could improve quality of life in patients with transfusion-dependent epistaxis [34].

Nasal closure of one or both sides, also known as Young’s procedure, is the only known option which has been shown to result in complete cessation of epistaxis. However, this has deleterious side effects, including chronic mouth breathing and partial or complete anosmia, which can compromise the quality of life. Thomson et al. evaluated the effect of nasal closure on quality of life [9]. They found that while nasal closure improved the ESS, it did not have a significant effect on sleep or quality of life, and must therefore be considered as a last resort. Another alternative or pre-evaluative option before complete nasal closure is the use of a nasal obturator. Woolford et al. described the use of a silastic nasal obturator in three patients, and observes that the frequency of epistaxis reduced on using the obturator [35].

### Limitations of the study

**Fig. 3:**
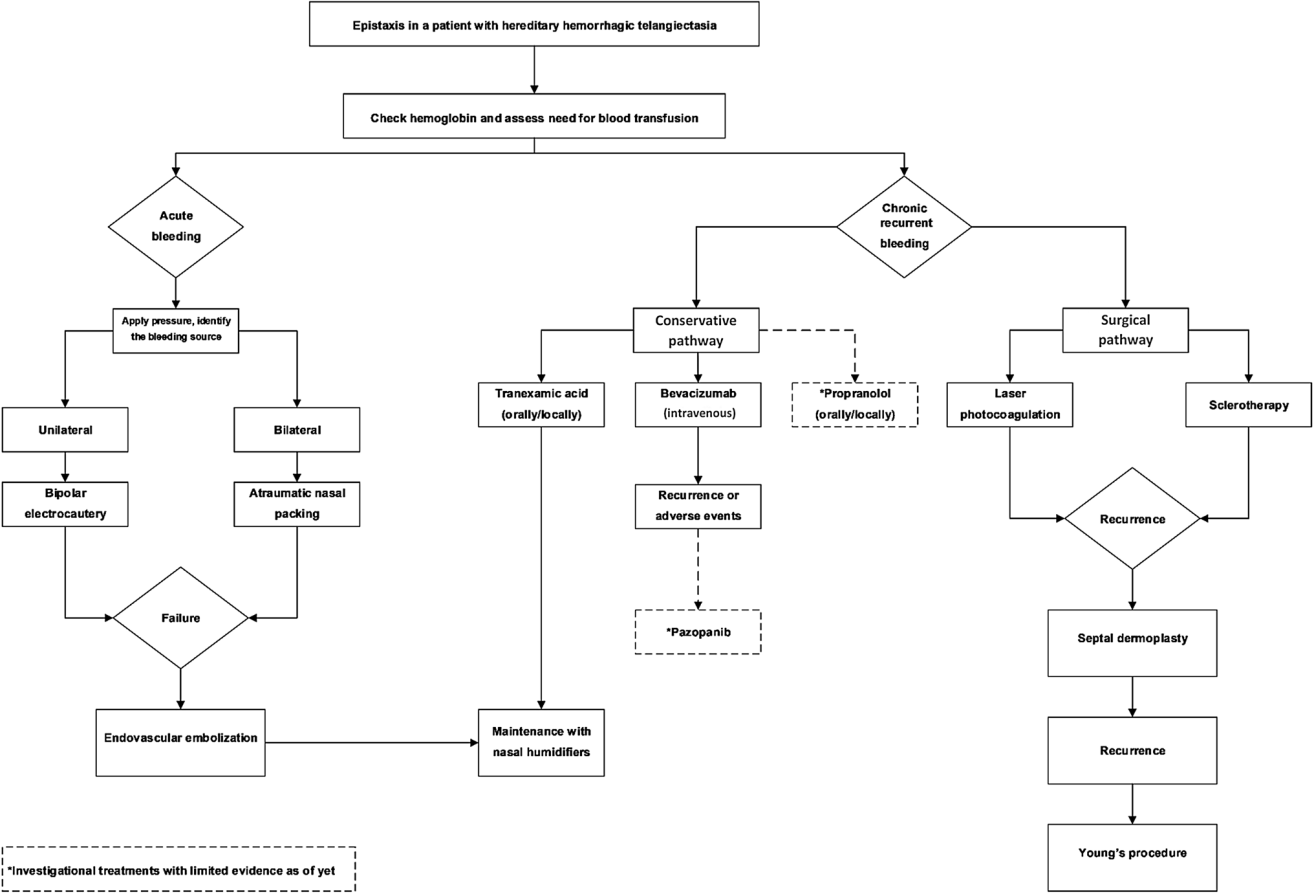
Proposed algorithm for the management of epistaxis in patients with HHT

One drawback of the study was that the primary outcome measure, that is, time until the next visit, is a patient-specific measure, dependent on disease severity, which has inter-individual variability. Although the current study measured the time to bleeding following each treatment modality, it could not evaluate the particular efficacy of each treatment. For this, a randomised prospective trial would be required. Based on the evidence available from literature and our own experience, we propose an algorithm for the management of patients with HHT who present with acute or recurrent epistaxis (Fig. 3).

## Conclusion

Despite the vast plethora of potential treatments available, complete control of epistaxis in HHT patients is difficult to achieve without compromising the quality of life. Newer treatment options have emerged but need validation. Further research is needed with certain forms of therapy, including pazopanib therapy and sclerotherapy. In general, it is best to begin with conservative and medical management and escalate to surgical intervention when needed. The algorithm accompanying this article aims to provide a comprehensive and stepwise management approach in controlling bleeding with minimal impact on patients’ quality of life.

## Data Availability

Available with corresponding author on request.
